# Handover of Critical Patients in Urgent Care and Emergency Settings: A Systematic Review of Validated Assessment Tools

**DOI:** 10.3390/jcm10245736

**Published:** 2021-12-08

**Authors:** Ruth Tortosa-Alted, Estrella Martínez-Segura, Marta Berenguer-Poblet, Sílvia Reverté-Villarroya

**Affiliations:** 1 Hospital de Tortosa Verge de la Cinta, Catalan Institute of Health, Pere Virgili Institute, Carretera Esplanetes, 14, 43500 Tortosa, Spain; rtortosa.ebre.ics@gencat.cat (R.T.-A.); silvia.reverte@urv.cat (S.R.-V.); 2 Nursing Department, Campus Terres de l’Ebre, Universitat Rovira i Virgili, Avenue Remolins, 13-15, 43500 Tortosa, Spain

**Keywords:** surveys and questionnaires, psychometrics, validity and reliability, patient handoff, emergency medical services, prehospital emergency care

## Abstract

The emergency handover of critical patients is used to describe the moment when responsibility for the care of a patient is transferred from one critical patient care healthcare team to another, requiring the accurate delivery of information. However, the literature provides few validated assessment tools for the transfer of critical patients in urgent care and emergency settings. To identify the available evaluation tools that assess the handover of critical patients in urgent and emergency care settings in addition to evaluations of their psychometric properties, a systematic review was carried out using PubMed, Scopus, Cinahl, Web of Science (WoS), and PsycINFO, in accordance with PRISMA guidelines. The quality of the studies was assessed using the COSMIN checklist. Finally, eight articles were identified, of which only three included validated tools for evaluating the handover of critical patients in emergency care. Content validity, construct validity, and internal consistency were the most studied psychometric properties. Three studies evaluated error and reliability, criterion validity, hypothesis testing, and sensitivity. None of them considered cross-cultural adaptation or the translation process. This systematic psychometric review shows the existing ambiguities in the handover of critically ill patients and the scarcity of validated evaluation tools. For all of these reasons, we consider it necessary to further investigate urgent care and emergency handover settings through the design and validation of an assessment tool.

## 1. Introduction

Today’s increasingly complex health system makes communication between healthcare professionals essential to guaranteeing quality, safety, and consistency across all levels of care [1,2,3,4]. In the urgent care and emergency field, the link between two levels of care for critically ill patients is called handover or handoff [5,6,7,8,9]. This is defined as the process that occurs when responsibility for the care of a patient is transferred from one critical patient care healthcare team to another, requiring the accurate delivery of information [10]. There are two types of transfer: temporary (shift change) and definitive (change of unit or level of care, or inter-hospital transfer) [11].

Handover is a current issue of concern for leading patient safety groups, as communication problems account for 60% of sentinel events reported to the Joint Commission, besides leading to increased costs and longer hospital stays [7,12,13]. It is important to note that, due to the characteristics of urgent and emergency care, the handover of critical patients usually takes place in a chaotic environment. It is also the only opportunity for professionals to exchange information [12,14], which aggravates communication issues among practitioners in the field. To address this phenomenon, in 2005, the World Health Organization (WHO) promoted the World Alliance for Patient Safety, in which it identified six areas of action, with one of them being communication during patient transfer [15,16]. In the same year, the Joint Commission on Accreditation of Healthcare Organizations (JCAHO) was designated as a WHO Collaborating Center on Patient Safety Solutions to initiate and coordinate the development and dissemination of solutions to reduce harms associated with healthcare. This gave rise to the Nine Solutions for Patient Safety, where the third solution addresses communication during patient transfer [16].

The available literature is limited, and there is great variability in the protocols, methods, and standards [17,18]. A lack of consensus on how to conduct handovers has led to heterogeneous literature and practical variability, hindering advances in the field [18]. In order to end ambiguity regarding the process of handover, an evaluation tool is needed to assess the different practices and methods available in a clinical setting in order to reach a theoretical and practical consensus.

A systematic review carried out in 2017 identified all of the handover assessment tools available in the literature from 2008 to 2015 [19]. In this review, Davis et al. [20] collected 32 assessment tools, of which only one, the dINAMO checklist [21], refers to the evaluation of handover in emergencies. This instrument has not been validated to date.

Thus, the literature provides few validated assessment tools for the transfer of critical patients in urgent care and emergency settings, despite handover being a subject of great importance that affects the clinical outcomes of patients. It is, therefore, crucial to expand and develop this line of research. The present review aims to identify validated evaluation tools in the literature that assess the handover of critically ill patients in emergency situations and to evaluate the psychometric properties of these tools.

### Objectives

The PICO mnemonic was used to formulate the research question:how many assessment tools on the handover of critical patients between professionals from different emergency departments are currently validated?

In order to answer this research question, we aimed (1) to identify validated emergency handover assessment tools in the literature and (2) to evaluate the psychometric properties of these tools.

## 2. Materials and Methods

### 2.1. Study Type

This systematic psychometric review was carried out in accordance with the preferred reporting items for systematic review and meta-analyses (PRISMA) [22]. The evaluation of the psychometric properties of the surveys and questionnaires was conducted with reference to the consensus-based standards for the selection of health measurement instruments checklist (COSMIN) [23]. The first point of the COSMIN checklist [23], general recommendations for designing a study on measurement properties, contains general standards that should be considered in the design of a study on any measurement property. The remaining items contain standards for specific studies on each of the 9 measurement properties: content validity, structural validity, internal consistency, cross-cultural validity/measurement invariance, reliability, measurement error, criterion validity, hypotheses testing for construct validity, and responsiveness [23]. Each property was scored on a 4-point scale with 4predefined options: very good, adequate, doubtful, and inadequate [23]. The overall score of a psychometric property was graded based on the worst-score-counts principle [23]. Psychometric properties that were not available in the published study were marked as not applicable (NA).

### 2.2. Selection Criteria

The inclusion criteria were studies dealing with tools for evaluating the handover of critical patients in the emergency setting that had passed the validation process. Only studies published in English and/or Spanish between January 2015 and May 2021 were included. 

Articles that did not describe the methodology or protocols used, bibliographic and systematic reviews, meta-analyses, and conferences were excluded. Furthermore, those that dealt with transfers in non-emergency contexts and those that did not indicate at least one psychometric property were also excluded.

### 2.3. Data Collection

This systematic review was carried out over May and June 2021 across 5 databases: PubMed, Scopus, Cinahl, Web of Science (WoS), and PsycINFO.

A combination of keywords used was medical subject headings (MeSH), and the subtopics or entry terms included “surveys and questionnaires”, “psychometrics”, “validity and reliability”, “patient handoff”, “emergency medical services”, and “prehospital emergency care”. The free keywords used included “assessment tools”, “instrument validation”, “handoff”, “handover”, “emergency”, and “prehospital”. The Boolean operators “AND” and “OR” were the basis of the search strategy used in each database (Table 1).

### 2.4. Data Analysis and Processing

**Figure 1:**
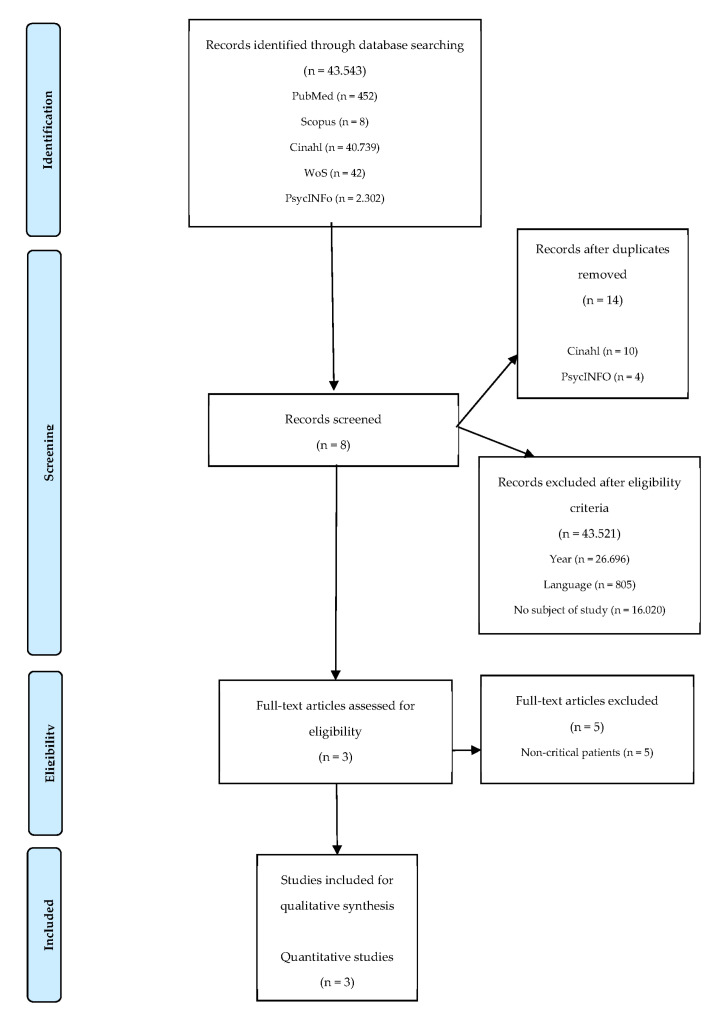
Literature screening and selection based on PRISMA flowchart.

The selection process was carried out following the 4 phases of the PRISMA flow diagram [22] (Figure 1).

In the first phase, the authors RTA, SRV, MBP, and EMS identified all of the available literature on the subject of study. In the second, the authors RTA, SRV, MBP, and EMS screened the results by applying filters and the inclusion-and-exclusion criteria, while eliminating duplicate citations. In the third phase, the authors RTA and SRV independently assessed the suitability of the selected articles, determining whether they met the objectives and inclusion criteria proposed. In case of disagreement, the opinions of the other 2reviewers, the authors MBP and EMS, were considered. Finally, in the fourth phase, the authors RTA, SRV, MBP, and EMS performed a careful reading of the selected articles. The authors were contacted in cases where a further inquiry was necessary.

The authors RTA and SRV independently assessed the methodological quality of the selected assessment tools using the COSMIN checklist [23]. In case of a discrepancy, a third author (MBP) was requested to review it.

The authors RTA and SRV extracted the following data from the articles to be included in the systematic psychometric review: general characteristics of the assessment tool (name, number of domains/factors and items, and response options), methodological characteristics (design, practitioners/respondents, size of sample, field of study, country and language), and the results of psychometric properties.

## 3. Results

The initial search generated 45,543 results. After eliminating those written prior to 2015, those not written in Spanish or English, and articles that did not deal with the handover of critical patients in an emergency setting, the results were narrowed to eight [24,25,26,27,28,29,30,31]. After exhaustive reading, five were excluded [25,26,27,28,30] as the handover did not take place in the study setting. Finally, three articles [24,29,31] were included in the systematic review. The PRISMA flow diagram graphically describes the results selection process (Figure 1).

In terms of geographic location, two of the studies were carried out in the United States and the other in Saudi Arabia (Table 2 and Table 3).

Content validity, construct validity, and internal consistency were the most common psychometric properties [24,29,31] (Table 3). Two of the studies assessed error and reliability, criterion validity, hypothesis testing, and sensitivity [24,31]. None of the studies appreciated cross-cultural adaptation or the translation process [24,29,31].

Each of the three [24,29,31] created a tool for evaluating the handover of critical patients in the emergency department while presenting a transferable framework or common scenario.

### 3.1. Assessment Tool 1: Standardization of Pediatric Emergency Handover through the Application of a Shared Mental Model

The aim of Sochet et al. [24] was to assess the face validity; to describe the sustainability of a handover standardization checklist for children aged 0–18 years admitted to the Pediatric Intensive Care Unit, the Neonatal Intensive Care Unit, and the Emergency Department of a tertiary referral hospital in the United States; and to compare their results with data obtained one year prior in a previous study.

The authors [24] divided their evaluation into three results. The first dealt with the shared mental model index (SMMi), expressed as the percentage of congruence among handover participants on the key patient health data included. This was measured using five short questions answered by the participants immediately after the handoff took place. The second outcome regarded face validity as assessed by participants’ perception of four items that were evaluated using a five-point Likert scale. The third involved handover sustainability and the team (participants), which were analyzed by qualitatively comparing the results collected with those obtained a year prior. The evaluation by Sochet et al. [24] consisted of 3 domains and 15 items.

- **Reliability:** internal consistency was not calculated. The temporal stability was of “doubtful” methodological quality [23], and it statistically showed a Kappa index of 0.92, indicating “very good” concordance with the tool [32].
- **Validity:** the methodological quality of content validity and hypothesis testing was “adequate” [23]. On the other hand, both criterion and construct validity were considered to be of “doubtful” methodological quality [23] due to poor statistical analysis of correlations and descriptions of item handling. Regarding sensitivity, the methodological quality was found to be “inadequate” [23] as there was no clear description of what happens during the interim period or possible changes in the study participants.

### 3.2. Assessment Tool 2: Emergency Medicine Handoff Tool

This tool was created by Alrajhi et al. [29] in Saudi Arabia by applying a Delphi method to identify the essential elements of handover in an emergency department. 

The Delphi method was carried out by 25 Saudi Arabian emergency physicians with more than three years of experience. It consisted of four stages [29]. In the first, they provided a list of all the items they considered relevant, and the panelist eliminated duplicates and generated a list organized into general domains. In the second stage, the physicians received the list of items in electronic format and were asked to rate each of the items according to their importance for emergency handover, using a 10-point Likert scale ranging from 1, “rarely required”, to 10, “always required”. The panelist calculated the mean of each item and summarized the experts’ arguments in addition to adding their own. In the third stage, the physicians received a spreadsheet with the group average compared with their own score for each item. In this phase, each expert had the opportunity to correct their score and/or comment to influence others in the group. In the fourth and final stage, the scores were recalculated based on stage three, and the comments were collected and sent to each expert. The final instrument consisted of 4 domains and 32 items [29].

- **Reliability:** Alrajhi et al. [29] evaluated internal consistency and temporal stability between stages. The internal consistency was rated as “very good” [23] and showeda Cronbach’s α of 0.93, considered “acceptable” [32]. The intraclass correlation coefficient (ICC) of 0.36 indicated a “poor” degree of agreement [32] for each of the stages.
- **Validity:** The methodological quality of content validity was “adequate” [23]. Construct validity, on the other hand, was considered “doubtful” [23]. Alrajih et al. [29] performed an exploratory factor analysis but did not provide a clear and extensive description of the analysis. In addition, they performed the analysis on a smaller-than-appropriate sample [23].

### 3.3. Assessment Tool 3: Cognitive Load Inventory for Handoff (CLIH)

The CLIH instrument [31] was designed by Young et al. [31] to measure the cognitive load experienced by participants during patient handovers. Cognitive load theory was originally developed by John Sweller in the context of studying student problem solving and was identified as a framework through which the cognitive mechanisms of transfer errors can be explored in order to develop new and more effective transfer strategies and protocols [31].

Cognitive load theory is derived from the concept of limited working memory for learning and is made up of three types of cognitive load, which is why the CLIH instrument is distributed in three domains, with a total of 16 items [31].

(1) Intrinsic load (IL), evaluated through five items, arises from the information processing demands associated with the performance of the task itself.
(2) Extraneous load (EL), evaluated through seven items, occurs when learners use working memory resources to process information not essential to the task.
(3) Germane load (GL), evaluated through four items, represents the information processing load caused by the learner’s deliberate use of cognitive strategies to refine existing schemata and to enhance storage in long-term memory.

- **Reliability**: the tool was considered “acceptable” [32] and of “very good” [23] methodological quality, with a Cronbach α of 0.85, 0.87, and 0.91 for the IL, EL, and GL, respectively [31]. It showed “doubtful” methodological quality [23] in the assessment of error and reliability due to the absence of an ICC calculation. However, Young [31] gauged this by applying Pearson’s correlation coefficient between IL, EL, and GL and their respective items, obtaining moderately strong scores for IL (0.51) and EL (0.75), though not for GL (0.22) [32].
- **Validity:** both content and construct validity were of “very good” methodological quality [23]. Young [31] applied an exploratory factor analysis (EFA) and a confirmatory factor analysis (CFA), obtaining optimal and statistically significant results (EFA: 0.52–0.90 for IL, 0.40–0.75 for EL, 0.50–0.86 for GL; AFC: 4.76 ± 2.06 for IL, 2.65 ± 1.88 for EL, 3.45 ± 2.29 for GL) [31].

## 4. Discussion

Most handover evaluation tools available in the literature assess transfers that occur in non-emergency settings, where the patient is not in a critical condition [25,26,27,28,30]. On the other hand, most of the instruments identified that do evaluate handover in urgent care and emergency settings do not validate their application in that field and are, therefore, not considered validated assessment tools [21,33,34].

The methodological quality of the psychometric properties of the tools included in this review was determined using the COSMIN checklist [23]. While the tools presented by Sochet et al. [24] and Young et al. [31] comply with 7 of the 10 COSMIN items [23], Alrajhi et al. [29] only complywith 3. It should be noted that COSMIN [23] is a very strict tool in which the final score obtained in each of the sections is determined by the lowest value of the items evaluated. Therefore, non-compliance with even a single factor already ranks the tool in a lower category [23]. For this reason, the ranking of the methodological quality of the articles which validate the handover assessment tools analyzed in this review is doubtful.

The instrument by Alrajhi et al. [29], the Emergency Medicine Handoff Tool, is divided into four domains: “non-clinical patient information”, “clinical patient information”, “evolution of the patient in the emergency department”, and “general condition of the patient in the emergency department”. By contrast, Sochet et al. [24] divided their tool into three domains: “shared mental model index (SMMi)”,“perception”, and “transfer and team”. Young [31] divided his instrument into three domains: “intrinsic load”, “extraneous load”, and “germane load”. As a whole, these authors have taken into account a wide variety of items to assess handovers in emergency medicine.

Regarding the items and factors that make up the tools, there was some discrepancy with the recommendations of the WHO and JCAHO to improve communication between professionals in the WHO Patient Safety Solutions [15,16]. Both Alrajhi et al. [29] and Sochet et al. [24] assessed only patient identification, while experts point to the correct identification of professionals as essential to guaranteeing proper transmission of information [5,15,16,18].

Nevertheless, there were also items in the aforementioned studies that the WHO and JCAHO [15,16] consider important elements for the transfer of information. Experts confirm that the optimal location for the transfer is the bedside [8]. Alrajhi [29] mentions the location where the information was transferred through the item “localization” within the “non-clinical patient information” domain.

Another relevant aspect for experts is the field in which the information is transferred. Ebben [35] describes a “good handover” as quiet, respectful, and organized, whereas Zakrison [18] explains that a “difficult handover” is chaotic and noisy, with multiple interruptions and with no clear leadership. Both Sochet [24] and Young [31] considered the environment in which information was transferred. Sochet [24] evaluates it through the item “degree of interruptions/distractions”, and Young [31] assesses it in the EL domain through the items “I was frequently interrupted” and “noise made it difficult to concentrate”.

Young’s instrument [31] also assesses what experts know as professional behavior, and San-Juan Quiles [5] reports that individual behavior during handover proved to be the key to the correct reception of messages. Young [31] includes this section in the EL domain through the items “the other clinician used jargon out of context”, “I was self-conscious due to who was present”, “I was thinking about things unrelated to the sign-out”, and “I found it difficult to focus my attention on the handover”. However, neither Sochet [24] nor Alrajhi [29] mentioned this section while discussing their instruments.

None of the three articles contemplates the standardization of communication in any of its domains or items [24,29,31]. Nevertheless, the totality of the literature ensures that the standardization of communication during the transfer is the best method of information transfer in addition to being associated with reduced errors and improved patient safety [5,12,13,18,33,34,36,37].

The three studies [24,29,31] evaluated the content of the handover using differing items and factors. This was probably due to the lack of consensus on handover procedures in urgent and emergency care [14,19]. It is essential that communication between two critical patient care teams is clear, specific, and concise [5,38], which reinforces the need for precision in content assessment during handoffs.

Other factors highlighted in the evaluation of the handovers by Sochet et al. [24] were efficiency and satisfaction. For Sochet [24], efficiency was represented by the mean handover durations determined by the initial time-out called by the emissor healthcare team and the cessation of handover discussion between providers. The efficiency of the handover was evaluated using a Likert scale, where the highest score was associated with a rapid handover [24]. However, we have not found a definition of an effective handover or the association of time spent on handover with efficacy. We also did not find out how much time should ideally be spent on a handover. 

Furthermore, while Sochet et al. [24] assessed overall satisfaction with the handover, reporting low/high responses [24], there was no clear definition of satisfaction or of what constitutes a successful handover [37,39]. On the other hand, Sochet et al. [24] scored the general satisfaction of the handover using a five-point Likert scale, in which 1 expresses strong disagreement and 5 strong agreement in receiving a satisfactory handoff. Although Young [31] does not consider the future treatment administered to the patient once received by the new care team, Sochet [24] and Alrajhi [29] established a plan for future care to guide the receiving team on the subsequent treatment of the patient. Sochet et al. [24] analyzed this through the item “anticipatory guidance”, while Alrajhi et al. [29] identified it as “care orientation”. Additionally, both studies [24,29] also considered the care offered to the patient during their treatment. Sochet et al. [24] differentiated between pre-and post-handover care, assessing actions during transport, and the post-admission care plan. Alrahji et al. [29] also considered an alternate plan and the identification of outstanding tasks for the patient’s receiving team. Alrajhi et al. [29] included the family of the patients in the evaluation of the handover. In particular, they considered the issues discussed with the patient and their family and the conflicts that may arise from them. Experts in the transfer of information confirmed that the ideal emergency handover involves healthcare professionals, patients, and relatives [40]. Involving patients and relatives in the process provides an opportunity to clarify unclear information and allows them to contribute additional information [40]. This approach decreases adverse events, improves communication, and enhances the continuity of patient care [40].

It is also worth noting that the tool in Sochet et al. [24] was validated only in critically ill pediatric patients and, although the scope of the study may be similar, its application in critically ill adult patients needs to be further explored.

The evaluation of handovers [31] through the calculation of the cognitive load of the people involved in the process, as proposed by Young et al. [31], is an innovative and novel approach. The application of CLIH [31] makes it possible to identify practices and strategies that improve learning, thus it would be interesting to take this into account in the development of new protocols for the handover of critical patients to help reduce some of the errors typical to the practice. However, CLIH [31] has only been applied to a sample of residents and fellows, thus we do not know what impact this instrument would have on the daily practice of emergency health professionals.

## 5. Conclusions

This systematic review reveals the existing ambiguities in the handover of critical patients in urgent care and emergency settings and the paucity of validated assessment tools to evaluate the process. In our review of the literature, we found only three validated assessment tools for the handover of critical patients in the emergency department, although each presented certain deficiencies in methodological quality and psychometric properties. In addition, we consider that some of the factors and items need to be reformulated as they diverge from expert recommendations, while others need to specifically frame and define the element described and how it is assessed. 

For all of these reasons, we consider it necessary to further investigate the handover of critical patients in urgent care and emergency settings through the design and validation of a robust evaluation tool.

## Figures and Tables

**Table 1:** Search strategy *n* = 5; Tortosa, ESP, Spain, 2021.

Database	Search	*n*No Filter
PubMed	(((((((questionnaire) OR assessment tools) AND validation) AND psychometric) AND validity) AND reliability) AND emergency handoff) OR emergency handover	452
Scopus	((KEY (instrument) OR KEY (tool) AND KEY (validation))) AND ((KEY (handoff) OR KEY (handover) AND KEY (emergency) AND KEY (prehospital)))	0
	(KEY (instrument) OR KEY (tool) AND KEY (validation) AND KEY (handoff) OR KEY (handover) AND KEY (emergency))	1
	(KEY (instrument) OR KEY (tool) AND KEY (validation) AND KEY (handoff) OR KEY (handover))	7
Cinahl	instrument validation OR assessment tools AND handover OR handoff AND emergency	40,739
Web of Science (WoS)	TS = (instrument validation OR assessment tools) AND TS = (handoff OR handover) AND TS = (emergency)	42
PsycINFO	instrument validation OR assessment tools AND handoff OR handover AND emergency	2302

**Table 2:** General characteristics of the assessment tools.

Tool	Objective	Language	Methodology	Sample	Town/City	Scope	Factors/ Domains	Items	Response Options
Shared Mental Model index (SMMi) (Sochet, 2018, USA)	To show that greater degree of SMMi can be achieved by using simple checklist-based handover standardization and can enhance other quality outcomes.	English	Retrospective descriptive and comparative cohort study	*n* = 100 pre = 50 post = 50	Nurses, doctors, technicians: prehospital and hospital	PICU NICU EC	1-SMMi 2- Face Validity Assessment 3-Handover and teaming metrics	5 4 6	Likert Scale (0 to 7- points) “I don’t know” rated as 0
								15	
Emergency Medicine Handoff Tool (Alrajhi, 2019, Saudi Arabia)	To identify the core elements essential for an emergency department and to developing standardized handoff tools.	English	Delphi	*n* = 25	MS Doctors	EM	1-Non-clinical patient information 2-Clinical patient information 3-Emergency department course 4-Emergency department status	3 10 10 9	Likert scale (1 = rarely required) to (10 = always required)
								32	
Cognitive Load Inventory for Handoff (CLIH) (Young, 2020, USA)	To measure the cognitive load experienced by trainees during patient handovers.	English	Psychometric study—cross-sectional survey	*n* = 1807	Residents	In-patient ICU In-patient non-ICU Emergency department Ambulatory Perioperative setting	1-Intrinsic load 2-Extraenus load 3-Germane load	IL: 5 EL: 7 GM: 4	Likert scale (0 = strongly disagree) to (10 = strongly agree)
								16	

PICU: Pediatric Intensive Care Unit; NICU: Neonatal Intensive Care Unit; EC: Emergency Center; PED: Pediatric Emergency Department; ICC: Intraclass Correlation Coefficient; CVI-I: Content validity index each of the items; CVI-S: Content validity index regarding the whole scale; KMO: Kaiser–Meyer–Olkin.

**Table 3:** Methodological quality of the psychometric properties of the assessment tools: COSMIN (23). Tortosa, ESP, Spain, 2021.

Tool	General Recommendation	Content Validity	Construct Validity	Internal Consistency	Cross-Cultural Adaptation	Error and Reliability	Criterion Validity	Hypothesis Testing	Sensitivity	Translation Process
Shared Mental Model index (SMMi) (Sochet, 2018, USA)	Very good	Very good	Doubtful	Doubtful	NA	Doubtful	Doubtful	Adequate	Inadequate	NA
Emergency Medicine Handoff Tool (Alrajhi, 2019, Saudi Arabia)	Very good	Adequate	Doubtful	Very good	NA	NA	NA	NA	NA	NA
Cognitive Load Inventory for Handoff (CLIH) (Young, 2020, USA)	Very good	Very good	Very good	Very good	NA	Doubtful	Very good	Very good	Very good	NA

NA: not applicable.

## Data Availability

Not applicable.
